# Effects of the COVID-19 Pandemic on Livelihoods and Social Support Mechanisms in Selected Regions of Tanzania: A Qualitative Study

**DOI:** 10.24248/eahrj.v9i1.822

**Published:** 2025-09-30

**Authors:** Peter E. Mangesho, Mohamed Seif, Elizabeth H. Shayo, Leonard E.G. Mboera, Mark Urassa, Mtumwa Bakari, Esther Ngadaya, Blandina T. Mmbaga, Nahya Nassor, Giuliano Russo, Kevin Deane, David Mccoy

**Affiliations:** a National Institute for Medical Research, Amani Medical Research Centre, 21405, NIMR Road, Mbaramo-Muheza, Tanga, Tanzania; b National Institute for Medical Research, The Headquarters, Dar es Salaam, Tanzania; c SACIDS Foundation for One Health, Sokoine University of Agriculture, Morogoro, Tanzania; d National Institute for Medical Research, Mwanza Research Centre, Mwanza, Tanzania; e National Institute for Medical Research, Tanga Research Centre, Tanga, Tanzania; f Kilimanjaro Clinical Research Institute, Kilimanjaro Christian Medical Centre and KCMC University, Moshi, Tanzania; g Z–Zanzibar Health Research Institute, Binguni, Tunguu Kibaoni Road, Zanzibar, Tanzania; h Queen Mary University of London, Mile End Road, E1 4NS, London, United Kingdom; i Open University, Walton Hall, Milton Keynes, MK7 6AA, United Kingdom; j United Nations University, International Institute for Global Health, UNU-IIGH Building, Kuala Lumpur, Malaysia

## Abstract

**Background:**

The coronavirus disease 2019 (COVID-19) has contributed to massive disturbances in people's economic, social, and cultural affairs. Such experiences were brought about by the pandemic itself and the intervention measures put in place to contain the transmission of the disease. This study assessed the impacts of the COVID-19 pandemic on social and economic dimensions in selected regions of Tanzania.

**Methods:**

The study was conducted in Unguja, Pemba and Dar es Salaam, Tanzania, employing a qualitative design. Thirty four interviews were conducted with key informants at the national, regional and district levels, comprising influential people, leaders, and healthcare workers. A total of 14 focus group discussions with community members and health care workers were conducted. All data were thematically analysed.

**Results:**

Study participants revealed the main impact of COVID-19 to be on businesses and trade, with loss of wage earnings due to a reduction in touristic activities and flow from abroad of essential goods for trade. This, in turn, was reported to affect people's purchasing power to fend for themselves, causing food insecurity at the household level. Trading activities of essential goods such as sugar and cooking oil were affected due to the reduction of their importation, which in turn caused their rental prices to rise. Restrictions imposed on gatherings created an atmosphere of fear that harmed traditional forms of support in times of need.

**Conclusion:**

The findings from this study demonstrates how COVID-19 mitigation strategies significantly disrupted the social and economic fabric of the communities studied. We recommend that future pandemic preparedness plans should prioritize the implementation of comprehensive support programs for at-risk households and enact price controls on essential goods to protect the most vulnerable from economic hardship.

## BACKGROUND

The COVID-19 viral disease pandemic, first reported in China in 2019,^1^ has continued to compel people to re-examine their current lifestyles. It is a disease with almost similar consequences to recent and historical pandemics, including HIV, the Middle East Respiratory Syndrome (MERS), the Spanish Flu and the Ebola disease.^2^ Unlike these diseases, Workies and colleagues,^3^ have argued that COVID-19 effects have had far-reaching ramifications because world urbanization and travel levels were not as prominent in previous pandemics as they are today. Hence, their spread and associated risks were confined to manageable areas. However, just under two months after the first case was reported, the World Health Organization (WHO) declared COVID-19 as a pandemic of international concern and a public health emergency.^4^ Unlike other pandemics, the COVID-19 pandemic has contributed to massive disturbances to the economy and social aspects of people's lives around the globe.^3,5^ Evidence of the pandemic impacts on sub-Saharan countries such as Tanzania need documentation for future pandemic preparedness.

The first COVID-19 case in Africa was reported in Egypt on February 14, 2020.^6^ As of March 09, 2022, 47 countries in Africa had reported 8,117,030 cases and 169,559 deaths.^5^ These cases accounted for 1.80% of the 449,927,05 cases and 2.82% of the 6,016,097 deaths that were reported globally. Taking a global view, the African continent has been affected comparatively less. Most of the countries followed the WHO-prescribed non-pharmaceutical control measures updated from time to time.^6^ These included regular hand washing, wearing face masks, restricting urban public transport, and practising social distancing. Others involved prohibiting public gatherings, ordering curfew, homestays (working-from-home), nationwide closure of schools, lockdowns, and closure of business and national borders.^7–9^

Infectious disease epidemics have had various effects on different facets of people's lives and the functioning of countries. Epidemics have been shown to disrupt health systems, limiting the capacity to deal with routine health issues, affecting their accessibility, and compounding the outbreak itself. The Spanish flu of 1918, HIV/AIDS and Malaria are examples of the epidemics that have disrupted services for other communicable and non-communicable diseases,^9,10^ reproductive health services, mental health and affected health workers’ optimal performance.^10^ They have also been documented to affect people's health-seeking behaviours, cause an increase in deaths, and impede access to preventable health conditions.^11–13^ The COVID-19 pandemic is reported to have magnified health inequalities, having surfaced on the backdrop of pre-existing epidemics, thereby disproportionately affecting the poor.^14^

Many factors influenced these interruptions, including the imposed obligatory and strict implementation of the mitigation measures to contain the spread of the disease. The COVID-19 pandemic and its mitigation measures have also impacted different sectors of countries’ economies. These include consumption sectors, foreign exports and imports,^15^ labour markets, capital markets, and specific industries such as transportation and tourism.^16^ Due to globalisation, the high social and economic interconnectedness between countries, regions and continents means that the impact of the pandemic has been trans-global. As a result, direct impacts include income loss, disruption of agricultural production,^3,15,17^ a decline in tourism,^1,18–22^ and a reduction in informal sector employment.^23^ For instance, a few months after the onset of COVID-19, it was reported that the pandemic caused significant declines in income amongst both formal and informal sectors of the economy.^24^ The education sector was also not spared as school-going children and higher learning institutes were affected.^18–20^ The pandemic hurt local economies in African countries, including the dependable agricultural sector,^17^ and hence food insecurity.^3,15,21^ Evidence has also shown how epidemics can shift the direction of the economies from the formal to informal sectors.^22^

The impact of the COVID-19 policy response measures varied within individual countries around the globe.^3,4^ Tanzania adapted WHO COVID-19 mitigation measures and related policies to fit its own unique situation. During the three months following the report of the first case in March 2020, it focused on school closures, banning unnecessary gatherings, and partial enforcement of social distancing. ^19–20^ After the three months of implementation of these measures, some of them were relaxed, and others were promoted. The implementation of these measures and the promotion of others had effects on people's everyday livelihoods. This study was conducted to understand better the impact of COVID-19 and its control measures on the livelihoods of people in selected districts of Tanzania.

## METHODS

### Study Sites

The study was conducted on the Zanzibar islands, which are part of Tanzania, and on the Tanzania mainland in Dar es Salaam Region. Zanzibar is composed of two Islands of Unguja and Pemba with a population of approximately two million people.^23^ It has a total area of 2,462 Sq. km and tourism, spice farming and fishing are considered the main sectors of her economy. On the other hand, Dar es Salaam, is the largest City and financial hub of Tanzania with a population of over five million residents. The City is made up of five districts which include Kinondoni, Ilala, Ubungo, Temeke and Kigamboni. In each study area, one district was purposefully selected: Mjini Magharibi for Unguja, Chake Chake for Pemba and Ilala for Dar-es-Salaam. The rural-urban criterion was considered whereby Mjini Magharibi and Ilala represent the urban setting, while Chake Chake represents the rural setting. The selection of sites was based upon the differences in response measures and impacts on the mainland and in Zanzibar, while taking into account the dichotomy between rural and urban areas. More details of the site have been published in a previous related study.^19^

### Study Design

The study adopted a qualitative exploratory design by combining typical qualitative data collection methods to understand the effects of Covid-19 pandemic on livelihoods and social support mechanisms in selected districts in Tanzania. The study combined in-depth interviews and focus group discussions to elicit the various ways people experienced and affected by the pandemic in Tanzania.

### Sample Selection

In each district, one ward was purposefully selected for community interviews and Focus Group Discussions (FGDs). The study population selection considered inclusion of diverse categories of participants to ensure representation of different voices from the community and institutions directly and indirectly impacted with the pandemic. Participants included community members, community health volunteers, community leaders and influential people. In each ward, local leadership assisted in identifying and classifying participating households into wealthier, middle, and poor, hence, 20 households were selected to represent these three categories in each ward. At the national and district levels, we identified officials at the Ministry and local government, health care workers, and community health volunteers who were involved in the pandemic response. Recruitment of key informants and healthcare workers was done in collaboration with the national and district health research focal persons. These included the Ministry of Health officials (From relevant ministerial departments and sections), the Ministry of Education, multilateral and bilateral organizations and non-governmental organizations working in the health and livelihood sectors. Reproductive health coordinators and educational officers at the regional and district levels were also interviewed.

### Data Collection Methods

Three main methods of data collection were employed. These included FGDs and in-depth interviews (IDIs) with key informants using guides with open-ended questions, as well as semi-structured interviews. The interview and focus group idiscussion nterview guides were first pre-tested through role-play among the study enumerators and the investigators. Errors found were rectified. Thereafter, the tools were further tested through a small pilot study at a community in Dar es Salaam, where the actual study did not take place. The exercise assisted in addressing issues of language and topic understanding, flow of the questions, and generally improving enumerators’ skills. The study investigators subsequently improved the tools.

Both FGDs and interviews were conducted in Kiswahili, a language familiar to most Tanzanians and were audio recorded after obtaining permission from the participants. The FGDs with community members included adults and adolescents, divided into male and female groups. This division between the two genders was strategic to enhance freedom of expression. The in-depth interviews lasted between 45 and 60 minutes, while FGDs lasted between 90 and 120 minutes. The interview and FGDs guides were developed to capture household livelihood experiences during the pandemic, specifically regarding employment and income, cost of living, food and nutrition, sources of support received, and the applied coping mechanism to facilitate their living. Private and quiet places such as in community centre offices were selected for the interviews.

Thirty four (34) in-depth interviews with key informants, community influential and government leaders at different levels. They included participants from the National (8), Regional (2) District (11) and Community (13) level. In addition, 60 interviews were conducted with selected household members. At the district and community levels a total of 14 FGDs, comprising of 144 participants, each with at least 8 to 12 people, were conducted. Eleven FGDs were conducted with community members, comprising of adults and adolescent groups. Three FGDs were conducted with health care providers working in health care facilities.^19^

### Data Analysis

The recorded interviews and FGDs were transcribed verbatim and translated into English. Two investigators went through the transcripts to confirm the correctness of the transcription. A thematic deductive framework approach guided the data analysis activity, whereby a framework with pre-designed thematic areas was used to direct the coding exercise in the field.^24^ First, the researchers familiarised themselves with the interviews by recording any analytical notes, thoughts, and exciting impressions related to the study's objectives and the pre-designed codes. The data were read and re-read to unpack and record patterns, including similarities and differences from the initial codes. These further codes formed sub-themes that were coded under the initial predesigned categories.^24,25^ For example, the initial coding framework contained broad categories such as ‘Business and services’, ‘Household food security’ and ‘Social capital’. These main categories were further probed about the ‘what’, ‘how’, ‘why’ and ‘who’ (e.g. what and how businesses and services were affected and who was affected; how food security was a challenge and who was impacted; what forms of social capital were strained and how), which resulted into sub-categories/sub-themes. To present the results, some quotes were used to illustrate and validate the themes. The results in this paper focus on both the periods when the country partially imposed restrictions and when they were completely relaxed on June, 2020.^26^

### Ethics Considerations

The study was approved by the National Health Research Ethics Committee (Ref: NIMR/HQ/R.8a/Vol.IX/3742) and the Zanzibar Health Research Institute (Ref: NO. ZAHREC/03/FEB/2021/03). Permission to implement the study was sought from local regional and district authorities. Before consenting, all participants read (or it was read out to them) information about the study. They were informed about voluntary participation, confidentiality, and anonymity principles. Privacy was maintained throughout the interview process. Participants were also given the opportunity to ask questions and seek clarifications before agreeing to participate in the study. Consent was obtained through signing, or thumbprint for those who could not write.

## RESULTS

A total of 14 FGDs, 34 in-depth interviews and 60 household interviews were conducted making a total of 239 participants involved in this study. In both techniques, females accounted for the majority of the participants. Most of the participants were in the 18 to 45 years age category. The majority of the participants had ordinary secondary school education and over two-thirds were married. The majority were civil servants, primarily working in the health sector. Other occupations included sailors, carpenters, farmers, fishermen, security guards, traditional healers and drivers. (Table 1)

**TABLE 1:** Socio-Demographic Characteristics of the Participants by District

Variables	Chake Chake District	Mjini Magharibi District	Ilala District	Total
	N (%)	N (%)	N (%)	n (%)
Sex				
Female	50 (54.3%)	51 (48.6%)	26 (61.9%)	127 (53.1%)
Male	42 (45.7%)	54 (51.4%)	16 (38.1%)	112 (46.9%)
Age group (years)				
18–45	46 (50%)	53 (50.5%)	25 (59.5%)	124 (51.9%)
46–83	46 (50%)	52 (49.5%)	17 (40.5%)	115 (48.1%)
Education level				
Higher institution of learning	22 (22.9%)	39 (36.4%)	24 (60%)	85 (35.5%)
Ordinary level	41 (44.6%)	50 (46.7%)	52 (12.5%)	96 (40.2%)
Primary school	22 (23.9%)	16 (15%)	11 (27.5%)	49 (20.5%)
No Formal Education	7 (7.6%)	2 (1.9%)	0 (0%)	9 (3.8%)
Marital status				
Married	65 (70.7%)	74 (70.5)	28 (66.7%)	167 (69.9%)
Separated	0 (0%)	0 (0%)	1 (2.4%)	1 (0.4%)
Single	19 (20.7%)	23 (23.9%)	10 (23.8%)	52 (21.8%)
Widow	8 (8.7%)	8 (7.6%)	3 (7.1%)	19 (7.9%)
Occupation				
Civil service	35 (44.3%)	35 (39.3%)	41 (57.7%)	111 (46.4%)
Trading	11 (13.9%)	22 (24.7%)	11 (15.5%)	44 (18.4%)
Others	33 (41.8%)	32 (36.0%)	19 (26.8%)	84 (35.2%)

In this section, we first present the perceived effects on business and trade, we then show how these effects are linked to the availability of consumer goods and reduced money circulation. Thereafter, we report the effects of trade on prices of essential goods, which in turn affected household-level economic dynamics, including the loss of social capital. While these were reported as challenging outcomes, participants also divulged different coping strategies to mitigate challenges brought in by the pandemic.

### Effects on Businesses and Trade

Echoes of the decline of the tourism industry were heard in almost all the interviews and FGDs. During the COVID-19 pandemic, most of the hotels were closed due to the absence of international tourists. This resulted in the laying off and retrenchment of workers. Apart from the effect linked to hotel employment, others who were equally impacted by this were tour guides, shop owners, carving makers and sellers, and taxi operators. It was reported that while some employees were paid half salaries, others were put on unpaid leave, while others were laid off. The quote below typically represents the opinions of the participants:

“*The tourism sector employs the majority of Zanzibar's youth. The sector has been a source of income for both the government and individuals. For example, about 60% of the government's income comes from tourism. There are two types of employment here: those who are directly employed in hotels and those who are involved in small businesses selling various tourist items on the streets. All these businesses collapsed during the COVID pandemic*”.

(IDI, Ministry level, Mjini Magharibi)

The effects of COVID-19 were also felt by fisherfolks who depended on the ocean to make a living. They were also impacted as prices of sea products, including fish, dropped as a result of the collapse in tourism:

“*For example, most youth here depended on hotel jobs. Because the tourists were not coming, many hotels were closed. In addition, fisherfolk failed to sell their fish, such as fill fish and octopus, which had huge markets in the tourist hotels So all those lost their market since one kilo was supposed to sell for six thousand shillings (approximately 3 USD) dropped to three thousand shillings (approximately 1.2USD) Since it lacked sufficient markets, fish prices dropped in such a way that fisherfolk were affected*”.

(IDI, Regional Level, Chakechake).

The loss of jobs was most experienced during the initial phase of the pandemic when movement restrictions were imposed. The strict enforcement meant a sudden discontinuation of jobs, building construction tenders, and even potential openings and recruitments that were already being advertised:

“*During the COVID-19 all job advertisements and calls for interviews were cancelled because people were advised to stay at home as one of the preventive measures against COVID-19. COVID-19 caused 75% unemployment among the youths*”.

(IDI, District Level - Mjini Magharibi).

### Effects on the Availability of Goods

The restrictions on travel and trade imposed by foreign governments on their own countries, which are major trade partners with Tanzania, resulted in the poor availability of essential goods for trading. This was revealed in most community discussions and interviews at the household level. For example, countries like the United Arab Emirates and China were mentioned as the main trade partners with Tanzania. Goods are commonly shipped to the country and are a source of income for traders; as one community leader remarked during one of the FGDs, “*…no ship docked here, and people who depended on small businesses were highly affected*” (FGD, Community Leaders, Chake Chake). The failure deliver goods in time meant that the businessperson also lost their income and eventually depleted the capital needed to sustain their business in the future.

“*…As I told you earlier, Pemba is very dependent on foreign trade. Goods such as food and medicine come from outside the country, such as China and Dubai, as well as other countries. So the travel bans have led to shortages of some goods …*”.

(KII, District level, Chake Chake).

A similar situation was also experienced in Ilala. Study participants, throughout the interviews, mainly mentioned goods such as cooking oil and sugar:

“*… there was no way out to deal with the situation because even the business was so hard in the markets. Most of the big businesspersons did not go out to bring products. Most of the products were not brought here…*”.

(Household IDI Ilala).

The shortage of necessary products in the markets contributed to poor money circulation and affected traders’ restocking capacity. Traders reported situations whereby they were compelled to give customers goods on credit, which in turn affected their businesses, as this head of household in Ilala district accounted:

“*The situation is yet to get back to normal. This is 2021, and it is approaching 2022, but it will take time for the economy to return to normal. A number of people cannot pay cash for items they would like to buy from shops. As a result, they take the products with a promise to pay later when they get money, hence making the business unprofitable*”.

(Household interview Ilala).

### Effects on Customer Numbers, Money Circulation and Prices of Goods

The deficiency of general goods in the markets, coupled with COVID-19 restrictions, was reported to influence both the circulation of money and the availability of customers. Unlike in Mjini Magharibi and Chake Chake, where a portion of the population experienced loss of employment in most tourism sectors, a different experience was observed in Ilala. Petty traders, including street hawkers, tailors, food vendors (primarily women), reported a loss of customers whom they depended on for a daily income. However, in this case, participants reported that a massive decline in the number of customers was highly influenced by fear of contracting the virus as their operations involved gatherings and the inability of some customers to pay in cash following the loss of their daily incomes. Some of the petty traders decided to stop operating their business while waiting for the situation to be resolved:

“*The other challenge was that of staying at home. Sometimes, you were required to go to the market for business, but you can't go there for fear of contracting corona [sic]. Hence, you decide to stay at home*”.

(Household interview, Ilala).

For communities that depended on agriculture, the lack of cash was directly caused by the inability to sell their farm produce. The failure to sell goods affected their household purchasing power because of the lack of cash:

“*Household food security was low to a great extent due to economic decline and business failure. Those who depended on agriculture also failed to sell their goods because their customer did not have the money. Hence, the impact on food insecurity was so huge…*”.

(IDI, District level, Mjini Magharibi).

As a result of the shortage of essential goods due to border closure and travel restrictions, some respondents reported a tendency of some traders to hike the prices of goods available in the markets but had become scarce. The price hike indirectly raised the cost of living for some community members, affecting their livelihoods.

“ *yes, the prices of some products were high in the shops because they said we had to wait for borders to open. Some prices went up, others dropped. For example, it has been observed that until today, cooking oil is not only difficult to find but has also become too expensive….*”.

(Household interview, Ilala).

In Mjini Magharibi and Chake Chake, there were mixed reactions during the discussions on the rising prices of consumer goods. For example, while the cost of items such as sugar and oil were reported to rise, those of sea products such as fish declined, with participants citing the absence of tourists as the leading cause of this price drop:

“*Food prices went up, and the availability of food was limited. Farmers did not go to the market to sell their produce, and fishermen didn't go fishing. During the COVID-19 pandemic, fishermen were told to stay at home until the disease was over*”

(Household interview Chakechake).

### Effect on Household Food Security

This theme on household economies arose from discussions on how COVID-19 response measures impacted households. It was reported that the implementation of measures affected household food choices, nutrition and even changes in diet. The issue of food availability was prominently discussed when compared to the situation before the pandemic. Participants reported a change in their food habits, including rationing their food and sometimes changing their diets. This situation invariably affected the vulnerable age groups, especially children and the old:

“*People's choice of food was minimal. For example, here in Zanzibar, rice is the first choice, but people had to eat ugali (stiff porridge) because it was the only food available. Even children had no choice but to eat what was prepared at home*”.

(IDI, NGO representative, Mjini Magharibi).

It was reported that the frequency of drinking water increased during the pandemic. The increased frequency of water drinking was echoed as a coping strategy in the absence of food. The closure of schools exacerbated food demand and its resultant dynamics. Parents believed that they were not accustomed to having children at home, especially when they were supposed to be at school. This process seemed to interfere with usual household food economies. With reduced income, parents were compelled to do with the little they had, resulting in precarious situations:

“*As you know, if children are not at home, our food budget is low. But when the school was closed all the children were at home, the cost of living,, including food budget, increased*”

(Household interview Ilala).

There were, however, some households that had different experiences regarding food security. Food was not reported as a big issue because most of their meals were from their farm produce. What mattered was whether one farmed in that season or not, and if they did, whether they had enough stock to last them throughout the pandemic and its associated restrictions:

“*On our side, we have a small farm. Others have large areas of land where they grow different types of crops. We have never had food shortages. Some people grew maize and used their own maize stocks even during the COVID-19 pandemic. Those who did not have farms struggled in their own way to sustain life with their families*”.

(IDI, Community health volunteer, Chake Chake).

This situation differed in urban areas such as Mjini Magharibi and Ilala, where farms were unavailable, and people had to buy food from the shops and markets. All responses on household food availability in these two districts emphasized the need to exercise caution in planning daily consumption during the pandemic:

“*There was a shortage of food, and people did not have money to cover their family expenses, especially when the number of household members increased due to the school closure. Thus, whatever little we had was used carefully*”

(IDI, Influential person, Mjini Magharibi).

While the number of meal frequency was reduced to one or two, in some households, meals were totally missing:

“*Availability of food among those with low income was very challenging. People were staying without even being able to afford a single meal for their children*”.

(FGD, Community members, Chake Chake).

### COVID-19 Limitations on Social Capital

COVID-19 seemed to generate an atmosphere of fear and anxiety in and around the communities. The reports communicated fear of infection and caused subtle stigma towards individuals and families who were suspected of being infected by the disease. Households suffering from the disease had to deal with the disease on their own. Those who were not affected were equally restrained from offering support to those affected in fear of both reprisals from the rules against congregating, on the one hand, and getting infected themselves, on the other. As such, traditional social, economic and moral support became constrained. This atmosphere is well captured by this narration from a ministerial level health official:

*We have to accept that we were scared, and the stress killed us. There were a lot of cases where people died. As a doctor, I was scared too. Every time I checked the number of deaths in a day, it scared me. In other words, the first wave created fear, but the second wave, created an environment that isolated relatives*”

(KII, Ministry Level, Mjini Magharibi).

Extended stays at hospitals and in isolation centres impacted social life in general. Worse still, no one was allowed to visit patients either in the hospital or in the isolation camps. This was very new to most people, who used to visit their sick relatives in groups, but all these were discouraged during the pandemic.

“*Another thing that affected the family was the economy. When someone was sick, he or she was supposed to be isolated for 14 days. But in some families, these (sick) people were the bread-earners, hence isolating them for 14 days would mean starving their families*”

(FGD, Community Leader, Ilala).

Further, households that were not directly affected by the disease reported feeling stressed and depressed as they were compelled to stay at home and reduce movements, some of which were important to keep their households sufficient:

“*Yeah! People were so worried that they started feeling sick even though they were not. Every family was so worried. When a person had a fever, they thought it is COVID-19. Hence, every family member was supposed to stay at home and minimise movement. Inside the house itself, as soon as someone had a fever, other family members would thought it was corona [sic], causing lots of stress and depression among household members*”

(IDI, key informant Mjini Magharibi).

One household participant from Ilala commented that the imposition of limits to social interaction had a considerable effect on people's health in terms of their state of mind:

“*Restriction of movements has health impacts. We used to stay outside and meet with neighbours. But during this time, we stayed indoors and did not interact with other families. It was not good for our health. We were even affected mentally, because you couldn't interact with others…*”.

(Household interview Ilala).

Families and neighbours usually live while interacting daily by visiting their rural and urban houses, as families possess both urban and village houses. Visiting the village every week was a common sign of care. Parents and older household members preferred the rural area when COVID-19 came. However, most people were warned not to interact with their immediate family members regardless of the occasion, in fear of being branded as spreaders of the disease to their own kin:

“*Mmmh! I can't speak for other people, but as I said, there was tension in every household because nobody knew their fate. During that time, we were afraid to visit our neighbours or our parents in the villages. We did not visit them all. People were afraid of being seen as carriers of the disease. People thought that if I visited someone and he or she later got sick, I might be seen as the one who caused it*”.

(IDI, key informant Mjini Magharibi).

The common form of social capital benefit is exemplified and exhibited during family and communal ceremonies, especially weddings, burials, and self-help groups. COVID-19 restricted these gatherings. Relatives, friends and neighbours could not support their loved ones during burials to curb the spread of the disease. Traditionally, in the coastal communities, funeral ceremonies would last for days, as bereavement goes beyond economic support and includes moral and psychological support as well. However, families complained they were sometimes not allowed to bury their loved ones or, when they were, had to leave immediately after the burial, a directive that went against customs and norms:

“*After burials, people were supposed to leave. No gatherings were allowed. There were no family meetings to discuss post-burial mourning issues. Everything ended immediately after the burial. There was no mourning for the dead; once someone was buried, the business ended there. in most of the cases, people avoided crowds*”.

(Household interview Ilala).

Even after burial events had taken place, there was fear of visiting the deceased family. Such acts caused distress and a sense of isolation in both the families of the deceased and immediate social support groups. Describing an incidence, a household member had these to say:

“*They buried her husband somewhere I don't know where. But we never visited her, we were so afraid. It was until the day she came to our home when I told her that that this disease is dangerous, that is why we could not go back to offer our condolences*”.

(Household interview Ilala).

### COVID-19 Impact on Religious Practices.

In our study, the importance of fulfilling religious practices was powerfully demonstrated as a vital ingredient of what forms and holds a society together as people go about their daily lives. COVID-19 was reported to interfere with personal communication with God, a practice that fulfils an essential pillar of people's interaction with their God. For Muslims especially, most prayers, especially on Fridays, must be performed in a mosque in a gathering of fellow men, and worshipers would shake hands after prayers. Measures such as social distancing and isolation were reported as going against these essential practices. As a result, some people decided not to attend the prayers in the mosques and pray on their own at home. This was all due to the implementation of measures to reduce transmission at the community level, as was communicated by the authorities:

“*There was an impact on the community in general, especially for us believers. There were instructions, let's say, to keep a distance from another person while praying in the mosques. This directive was a strange one and challenging at the same time. The Imam (mosque leader) used to say the prayer rows should be full. then you were told to leave a space. Some of them agreed to, but others decided to pray at home*”.

(IDI, key informant Mjini Magharibi).

Furthermore, distortion of religious practices was reported when the deceased bodies were buried without even being washed, wrapped in a shroud, and prayed for.

“*The other thing which was very difficult to understand was the restrictions of people from attending burials or mourning and the use of covering a dead body with a shroud*”.

(IDI, key informant, Mjini Magharibi).

However, following the relaxation of the restriction measures, political leaders, especially the president, encouraged people to go about their daily lives. In that moment, prayers were strongly encouraged, and therefore, some of the religious buildings were reopened to allow the community to continue with their faith in the trying times of COVID-19. The congregation were required to adhere to preventive measures such as putting on face masks, hand washing, and keeping one-meter social distancing, which was challenging to implement in mosques. As a result, it was reported that the number of mosque-goers markedly declined.

“*People stopped going to the mosques, my husband and my brothers started praying inside the house, even the children did not go to the mosques anymore*”. (Household interview Chakechake).

## DISCUSSION

The government of Tanzania, like many other countries, instituted control measures to reduce and curb the spread of COVID-19. They included closures of all educational institutions, banning unnecessary gatherings, physical isolation or staying at home, wearing face masks, washing hands, and social distancing. Together with these, the imposition of lockdowns in other countries with direct and indirect links to Tanzania seems to pressure local communities’ ways of life and livelihood strategies. Results from our study reveal that businesses were greatly affected, which led to a scarcity of essential goods in the markets and reduced money circulation, which limited economic opportunities and survival options impacting household security especially that of food. Indirectly, traditional forms of social support were constrained out of fear of infection and socio-economic pressures in their households. Social, cultural, and religious practices, which are essential ingredients to individual and communal lives, were also greatly affected.

Just like in other tourist reliant economy countries,^21,27,28^ the reported effects of COVID-19 in Tanzania's tourism and allied sectors could result from over-dependence on income and revenues from a single industry that is highly interconnected to global markets. As a result, a ban on international flights,^29^ indirectly curtailed the flow of tourists to the country. The loss of jobs, the forced leaves, and insufficient flows of goods were inevitable. This shows that during pandemics, due to globalisation, it does not matter which part of the globe one resides in. Countries and their people will be affected disproportionately. Recent studies have shown that the recovery of tourism due to the COVID-19 pandemic is likely to take longer than expected due to its unpredictable recurring nature.^17,30^ Bailout and stimulus packages,^31,32^ innovative ways to “shock absorb” and to provide social protection to businesses and families,^33^ are highly needed in low-and middle-income countries such as Tanzania. This is important to deal with pressures emerging during pandemics.

Our results indicate how the marketplaces behaved in response to the pandemic as far as prices of goods, such as food products, were concerned. As people lost their jobs, their purchasing power also declined, while at the same time, some essential goods, such as sugar and cooking oil, became scarce. Dar es Salaam and Zanzibar markets rely on imported goods from outside the regions and country. Most likely, traders decided to increase food prices because of the uncertainty of the availability of new supplies, which could have kept them out of business for a long time. Another reason could be that they were taking advantage of shoppers who were purchasing goods out of panic in the fear that they would run out. The latter has also been reported from Ghana ^34^ and Pacific Island countries.^15^ The increase in prices during epidemics is not a characteristic of COVID-19 alone. Rising food prices were also reported during the Ebola virus disease outbreaks in Liberia and Sierra Leone.^35,36^ In countries whose economies are still growing, leaving the markets to readjust themselves may not be a viable option if the vulnerable are to be protected. Agreeable government interventions in market segments, such as pricing, may be necessary to prevent livelihood disruptions emanating from the behaviours of a few dishonest and greedy traders.

The loss of steady income due to COVID-19 and its control measures affected household food behaviours. The pandemic disrupted food security and consumption patterns, with the potential of dragging households further into poverty and malnutrition. The problem may have likely been worse in poor households, especially those with extended families. A similar study in Kenya reported how families opted for reduced meals by cooking less frequently and altering their diets. In Nigeria, more than half of respondents became food insecure because of COVID-19.^37^ Using a household dietary diversity score, a study in Bangladesh demonstrated how rural households received fewer quantities and types of food during the pandemic.^36^ However, Ethiopia, which did not go into total lockdown like Tanzania, shifted the pattern of consumption to a preference for staples and away from vegetables.^38^ Travel restrictions imposed during COVID-19 are likely to cause more harm considering that the majority survive on daily incomes and day-to-day activities, which are sometimes challenging to come by.^21,39^ In such situations, others have recommended emergency programs, including cash transfers to household women, in-kind voucher schemes and food feeding programs to provide social protection to vulnerable children.^17,33,39^ Further, governments could also plan and ensure that internal food supplies, such as promoting agricultural production in farming communities, are safely maintained.

In low and middle-income countries, the role of social protection systems in times of uncertainty, distress, and worry caused by emergencies such as pandemics is a valuable ingredient to coping strategies for the affected. ^40^ It is used in the absence of robust social security systems. During the pandemic in Tanzania, these social networks were, in one way, strained following the imposed restrictions on movements and social interactions. In Tanzania, normally, communities and households have been relying on each other specifically, in times of need.^41,42^ As a result, basic needs were complex to fulfil. During the early days of the pandemic in China, a study reported high psychological distress and less social support needed psychological intervention. In contrast, active coping strategies and increased social support were linked with decreased psychological distress.^41^ Almost similar results have been reported in Egypt and Iran.^30,31^

A big feature of the COVID-19 pandemic globally and in Tanzania was the reported number of deaths and the experiences associated with mourning the deaths. Our study revealed how community members struggled to come to terms with restrictions to participate in funeral ceremonies. Customary bereavement practices were being tested. The disease has already been reported to change the mourning landscape in Africa, whereby the elaborated nature of its funeral ceremonies is threatened.^43^ Generally, attending a funeral ceremony is a sign of showing respect to the dead and offering support to the family of the deceased.^44,45^ Failure to attend such ceremonies is a sign of self-isolation from the community. However, during COVID-19, all these were changed to reduce the risk of transmission. Other innovative ways of supporting families that have lost their loved ones are needed to protect local culture and religious practices while the dead are buried dignifiedly.

### Study Limitations

The study has reported experiences of COVID-19 and related measures on people's livelihoods and social support mechanisms in Tanzania. However, there are a few limitations that need to be highlighted. The data collection did not involve individuals in all major production sectors. However, we believe the results offer a relevant picture of what transpired regarding lived experiences of COVID-19 in communities and offer a starting point for future investigations on the actual impact of the pandemic on the economy of the country.

## CONCLUSION

This article set out to document the impacts of COVID-19 on livelihoods and social forms of support systems among selected communities in the coastal area of Tanzania. The findings of this study demonstrate how the COVID-19 pandemic created social and economic challenges as governments across the globe, including Tanzania, sought to curtail the spread of the disease through various control measures. Both urban and rural dwellers struggled to make ends meet following the loss of their income caused by the closure of businesses, which invariably affected their purchasing power. Further, hiked prices of essential goods resulted in household strategies in food rationing and reducing essential nutrition intake. Traditional forms of support were weakened following congregations’ restrictions, affecting livelihoods and traditional forms of support in times of need, such as burials. In the future, we recommend support programs that offer assistance to vulnerable households. Furthermore, the government should control the prices of essential goods to shield poor households from falling further into poverty.
